# The durable resistance gene *Tm-2*^*2*^ remains partially resistant to tomato brown rugose fruit virus

**DOI:** 10.1371/journal.ppat.1014380

**Published:** 2026-07-17

**Authors:** Shaokang Zhang, Mark A. Bernards, Aiming Wang

**Affiliations:** 1 London Research and Development Centre, Agriculture and Agri-Food Canada, London, Ontario, Canada; 2 Department of Biology, Western University, London, Ontario, Canada; University of Florida, UNITED STATES OF AMERICA

## Abstract

The tomato *Tm-2*^*2*^ gene is a highly effective, and durable resistance gene in agriculture that has protected tomato production against viruses of the *Tobamovirus* genus, such as tomato mosaic virus (ToMV) and tobacco mosaic virus (TMV) for over 60 years. This dominant *R* gene, originally sourced from wild tomato species (*Solanum peruvianum*), acts by recognizing the viral movement protein (MP) and triggering an immune response, often resulting in extreme resistance (ER). However, this durable protection is challenged by a recently emerged new tobamovirus named tomato brown rugose fruit virus (ToBRFV, *Tobamovirus fructirugosum*). ToBRFV-encoded MP is responsible for ER breakdown. Here, we present evidence that while ToBRFV can evade *Tm-2*^*2*^-mediated ER, *Nicotiana benthamiana* and tomato plants carrying *Tm-2*^*2*^ still remain partially resistant to ToBRFV. We show that ToBRFV MP is recognized by and interacts with Tm-2^2^ to trigger an attenuated hypersensitive response. Moreover, we discover that overexpression of Tm-2^2^ can enhance resistance to ToBRFV. These findings demonstrate the practical value of *Tm-2*^*2*^ in ongoing resistance breeding programs and open a potential avenue to restore *Tm-2*^*2*^ immunity through upregulation of *Tm-2*^*2*^ expression.

## Author summary

The *Tm-2²* gene is considered one of the most durable and effective genetic solutions for controlling tobamoviruses over the past six decades. However, a recently emerged new tobamovirus named tomato brown rugose fruit virus (ToBRFV) breaks down *Tm-2*^*2*^-mediated extreme resistance. Here, we report that while ToBRFV can evade *Tm-2*^*2*^-confereed ER, *Nicotiana benthamiana* and tomato plants carrying *Tm-2*^*2*^ still show partial resistance to ToBRFV. This attenuated resistance can be enhanced by upregulation of Tm-2^2^ expression. We uncover that Tm-2^2^ interacts with ToBRFV-encoded movement protein to trigger distinct, albeit attenuated, programmed cell death. These findings affirm the value of *Tm-2²* in tomato antiviral breeding, redefine *Tm-2*^*2*^ as a dose-sensitive, partially effective resistance gene against ToBRFV, and offers a possibility to restore *Tm-2*^*2*^-mediated immunity through expression enhancement.

## Introduction

During the recent global outbreak of COVID-19, a devastating plant-infecting positive-sense RNA virus named tomato brown rugose fruit virus (ToBRFV, *Tobamovirus fructirugosum*) also quickly spread worldwide [1–3]. The virus belongs to the genus *Tobamovirus* and naturally infects tomato and pepper with tomato as the primary host, Like other tobamoviruses such as tobacco mosaic virus (TMV) and tomato mosaic virus (ToMV), ToBRFV is extremely stable, and readily transmissible without the involvement of vectors. Infection by ToBRFV typically causes 30% - 50% yield losses, and disease outbreaks often result in the complete failure and early termination of the crop [1–3]. In view of its rapid, global spread and severe destruction, ToBRFV is metaphorically described as the plant version of COVID-19.

Tomato is the second most-produced and consumed vegetable in the world after potato. Among over 300 known viral species that affect tomato production, tobamoviruses, specifically TMV and ToMV, are considered major, persistent threats [3, 4]. In the past six decades, management of tobamoviruses heavily relied on the use of *Tm-2*^*2*^, one of the most durable resistance genes in agricultural history, which confers extreme resistance (ER, immunity) [5]. Tm-2^2^ belongs to the class of the nucleotide binding (NB) leucine-rich repeat (LRR) immune receptors, and acts by recognition of tobamoviral movement proteins (MPs) at the plasma membrane [6]. A conserved cysteine residue (C68) in TMV and ToMV MPs is crucial for both Tm-2^2^ recognition and viral cell-to-cell movement [7], creating a high fitness penalty for resistance breaking as mutations in the MP that allow the virus to escape Tm-2^2^ surveillance severely impair its ability to move intercellularly. Upon the Tm-2^2^ and MP interaction, Tm-2^2^ undergoes conformational change to promote its self-association [8]. The oligomerization of the Tm-2^2^ coiled-coil domain activates the hypersensitive response (HR) or programmed cell death to restrict infection [8]. Unfortunately, ToBRFV has evolved the ability to bypass *Tm-2*^*2*^-conferred ER, and ToBRFV-MP is responsible for resistance breakdown [9, 10]. To stakeholders, an urgent question is whether *Tm-2*^*2*^ still offers any protection (while not at the ER level) against ToBRFV.

To answer this inquiry, we carefully assessed ToBRFV infection in *Tm-2*^*2*^-carrying model and natural host plants and also examined if Tm-2^2^ recognizes ToBRFV-MP. We further explored the possibility to enhance Tm-2^2^-mediated immunity against ToBRFV.

## Results

### ToBRFV infection alters *Tm-2*^*2*^ transcription

**Fig 1:**
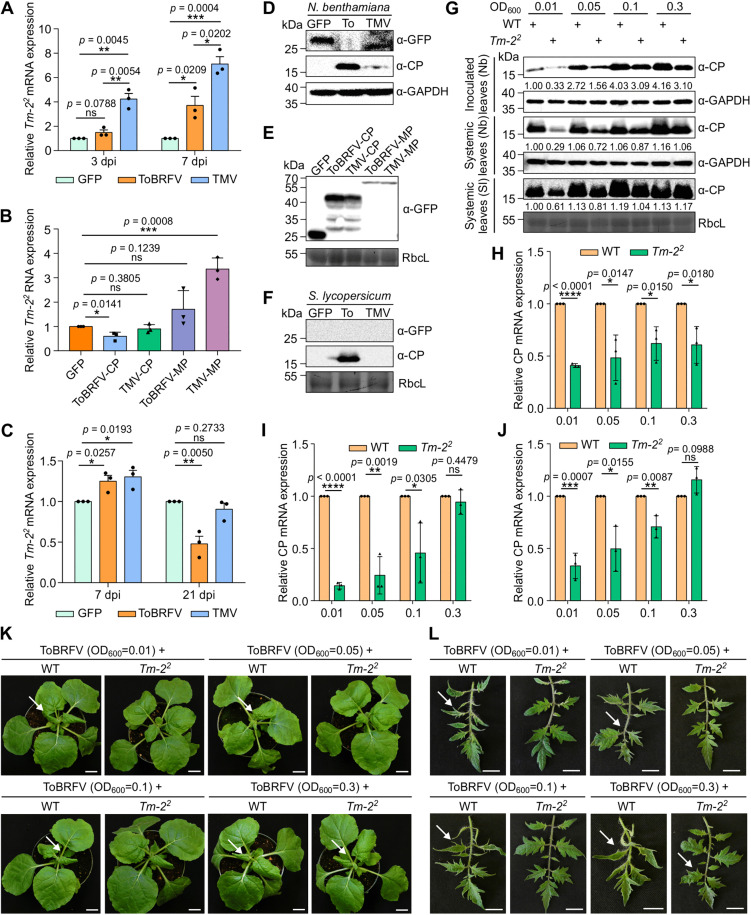
*Tm-2*^*2*^ confers partial resistance to ToBRFV. **(A and C)** RT-qPCR analysis of *Tm-2*^*2*^ transcript levels in *Tm-2*^*2*^-expressing *N. benthamiana* (A) and tomato (C) plants infected with ToBRFV at indicated time points. **(B)** RT-qPCR detection of *Tm-2*^*2*^ mRNA expression levels in *Tm-2*^*2*^ transgenic *N. benthamiana* leaves expressing GFP, ToBRFV-CP, TMV-CP, ToBRFV-MP, or TMV-MP at 3 dpi. **(D and F)** Immunoblotting analyses of viral CP and GFP in *N. benthamiana* (D) and tomato (F) plants. GAPDH (for *N. benthamiana*) or Rubisco large subunit (RbcL, for tomato) served as a loading control. **(E)** Confirmation of the protein expression in (B) by immunoblotting. **(G)** Immunoblot analysis of CP accumulation in wild-type and *Tm-2*^*2*^ *N. benthamiana* or tomato plants. **(H-J)** Relative viral CP mRNA levels in wild-type or *Tm-2*^*2*^-carrying *N. benthamiana* inoculated leaves at 3 days post inoculation (dpi, H) and systemic leaves at 5 dpi **(I)**, and Moneymaker tomato systemic leaves at 21 dpi **(J)**. **(K and L)** Phenotypes of plants inoculated with ToBRFV. Photographs were taken at 5 dpi for *N. benthamiana* (K) and 21 dpi for tomato (L) plants (Scale bars, 2 cm.). (A-C and H-J) *NbEF1a* and *SlEF1a* were used as internal controls for *N. benthamiana* and tomato plants, respectively. The data were analyzed using unpaired Student’s *t* test (*p < 0.05; **p < 0.01; ***p < 0.001; ****p < 0.0001; ns, not significant). All photographs were taken by the authors.

To probe the interplay between Tm-2^2^ and ToBRFV, we first investigated the impact of ToBRFV infection on *Tm-2*^*2*^ expression by inoculating transgenic *Nicotiana benthamiana* carrying *Tm-2*^*2*^ (driven by its native promoter) [11] with TMV (as a control) and ToBRFV full-length cDNA infectious clones [12, 13]. Real-time quantitative reverse transcription PCR (RT-qPCR) analysis revealed that TMV infection significantly induced *Tm-2*^*2*^ mRNA expression at 3 days post inoculation (dpi) in inoculated (local) leaves and 7 dpi in upper (systemic) leaves. ToBRFV infection slightly (insignificantly) stimulated *Tm-2*^*2*^ expression at 3 dpi, but significantly at 7 dpi, although to a lesser extent in comparison with TMV infection (Fig 1A). To examine if MP and coat protein (CP) of TMV and ToBRFV are involved in upregulation of *Tm-2*^*2*^, we conducted agroinfiltration to transiently express these proteins in *Tm-2*^*2*^ transgenic *N. benthamiana*, with the green fluorescent protein (GFP) as a control. Expression of TMV-MP and ToBRFV-MP stimulated *Tm-2*^*2*^ expression but only TMV-MP expression did at the significant level (Fig 1B). Surprisingly, ToBRFV-CP rather than TMV-CP suppressed *Tm-2*^*2*^ mRNA accumulation at 3 dpi. We further checked *Tm-2*^*2*^ expression in tomato plants (cv. “Moneymaker”) upon ToBRFV and TMV infection. As shown in Fig 1C, infection by ToBRFV or TMV upregulated *Tm-2*^*2*^ expression at 7 dpi. Along with ToBRFV infection progression, *Tm-2*^*2*^ expression was repressed at 21 dpi. In contrast, at this time point, no significant difference in *Tm-2*^*2*^ expression was found between GFP- and TMV-inoculated tomato plants. The expression of the abovementioned viruses and proteins was verified by immunoblotting analysis (Fig 1D–1F).

### *Tm-2*^*2*^ confers partial resistance to ToBRFV in a dose-dependent manner

The dynamic expression pattern of *Tm-2*^*2*^ in response to ToBRFV infection in both *N. benthamiana* and tomato (Fig 1A–1C) raises a possibility that *Tm-2*^*2*^ may still confer partial resistance to ToBRFV. To test this assumption, we inoculated *N. benthamiana* and tomato plants carrying or without *Tm-2*^*2*^ with different ToBRFV titers via infiltration of four *Agrobacterium* concentrations (OD_600_: 0.01, 0.05, 0.1 and 0.3). In the inoculated leaves (with all inoculum doses), fewer amounts of ToBRFV (at both protein and RNA levels) were detected in *Tm-2*^*2*^ *N. benthamiana* than the wild-type (Fig 1G and 1H). In the systemic leaves of *N. benthamiana*, this significant difference was also observed under relatively lower ToBRFV inocula (OD_600_: 0.01, 0.05 and 0.1) but not under high dosage (OD_600_: 0.3; Fig 1G and 1I). This also held true in the systemic leaves of tomato (Fig 1G and 1J). Consistently, *Tm-2*^*2*^ *N. benthamiana* and tomato with lower inocula exhibited milder symptoms than corresponding wild-type plants (Fig 1K and 1L). Similar results were also observed when mechanical inoculation was used for the assay (S1 Fig). These data strongly suggest that *Tm-2*^*2*^ confers partial resistance to ToBRFV.

To investigate if systemic acquired resistance (SAR), an induced immune mechanism in plants is involved, we assessed the expression of three SAR marker genes *Flavin-dependent Monooxygenase 1* (*NbFMO1*), *Isochorismate Synthase 1* (*NbICS1*) and *Nonexpressor of Pathogenesis-Related Genes 1* (*NbNPR1*). We found that ToBRFV infection significantly induced the expression of *NbFMO1* and *NbNPR1* in both wild-type and *Tm-2*^*2*^ *N. benthamiana* plants, indicative of the activation of SAR-related responses (S2 Fig). However, no significant difference was found in their expression levels between ToBRFV-infected wild-type and *Tm-2*^*2*^ *N. benthamiana*. ToBRFV infection slightly and insignificantly elicited the expression of *NbICS1*, the key gene involved in salicylic acid biosynthesis (S2 Fig). These results suggest SAR unlikely plays an important role in *Tm-2*^*2*^-mediated resistance, consistent with the previous finding that different strengths of *Tm-2*^*2*^-mediated resistance are not correlated with *Pathogenesis-Related Protein 1a* (NbPR1a) transcript levels [11].

**Fig 2:**
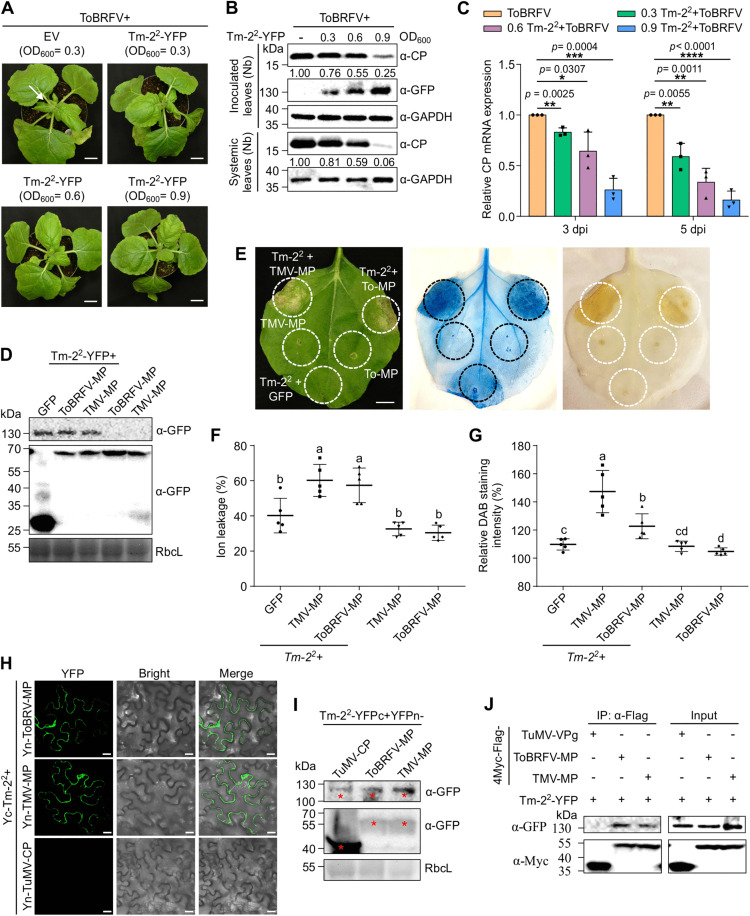
Tm-2^2^ interacts with ToBRFV-MP and triggers attenuated hypersensitive response. **(A)** Representative viral symptoms in wild-type *N. benthamiana* co-infiltrated with ToBRFV and increasing concentration of Tm-2^2^-YFP protein. Images were taken at 5 dpi (Scale bars, 2 cm). **(B)** Immunoblotting analysis of the effect of increasing concentrations of Tm-2^2^-YFP on ToBRFV-CP accumulation. **(C)** Viral CP transcript levels in inoculated and systemic leaves in *N. benthamiana*. **(D)** Protein expression was validated by immunoblotting. **(E)** Attenuated cell death phenotype (left and middle panels) triggered by transient co-overexpression of ToBRFV-MP and Tm-2^2^ at 3 dpi (Scale bar, 1 cm). H_2_O_2_ accumulations were visualized by 3, 3’-diaminobenzidine (DAB) staining (right panel). **(F and G)** Electrolyte leakage assays quantifying ion conductivity (F) and quantification of DAB-stained leaf patches (G) in **D.** Comparison analysis was performed using student’s *t* test (n = 5). Different letters indicate statistically significant differences. Different letters indicate statistically significant differences. **(H)** BiFC assays detecting the association between ToBRFV-MP and Tm-2^2^. TuMV-CP and TMV-MP served as a negative control and positive control, respectively (Scale bars, 20 μm). **(I)** Expression of indicated proteins in **(H)**. Red asterisks indicate the corresponding bands. **(J)** Co-immunoprecipitation analysis confirming the association between ToBRFV-MP and Tm-2^2^ in *planta*. All photographs were taken by the authors.

Since *Tm-2*^*2*^-mediated resistance to TMV depends on its expression level [11], we examined if this is also the case for ToBRFV. Tm-2^2^-YFP was transiently expressed in *N. benthamiana* plants at different levels through agroinfiltration (*Agrobacterium* OD_600_: 0.3, 0.6 and 0.9), followed by agroinoculation with ToBRFV. Clearly, overexpression of Tm-2^2^ inhibited viral accumulation in local leaves at 3 dpi and systemic leaves at 5 dpi, accompanied by reduced symptom severity (Fig 2A-2C). Under higher levels of Tm-2^2^ (OD_600_: 0.6 and 0.9), this inhibitory effect was more prominent, consistent with a dose-dependent pattern observed previously against TMV [11]. Consistent results were also obtained when ToBRFV was mechanically inoculated (S3 Fig).

### ToBRFV-MP associates with Tm-2^2^ and induces attenuated cell death

Given that *Tm-2*^*2*^ confers attenuated resistance to ToBRFV, one would ask if Tm-2^2^ recognizes ToBRFV-MP to activate the HR. A recent study showed that different from TMV-MP, ToBRFV-MP failed to induce cell death in *Tm-2*^*2*^ transgenic *N. benthamiana* [11]. To test if this difference is attributed to *Tm-2*^*2*^ expression profiles, TMV-MP and ToBRFV-MP were transiently expressed alone or co-expressed with Tm-2^2^-YFP at a relatively high concentration (OD_600_: 0.6) in wild-type *N. benthamiana*. Immunoblotting confirmed the expression of corresponding proteins (Fig 2D). As expected, co-overexpression of Tm-2^2^ with TMV-MP induced strong HR. A relatively weaker HR was evident in the leaf tissue co-overexpressing ToBRFV-MP and Tm-2^2^ (Fig 2E). This visual observation was further corroborated by trypan blue and DAB (3, 3′-diaminobenzidine) staining, and subsequent quantification analyses (Fig 2E–2G). These two histochemical stains allow for detecting cell death by visualization of hydrogen peroxide (H_2_O_2_) production or labeling dead cells exclusively [14].

To investigate whether Tm-2^2^ associates with ToBRFV-MP, we performed a bimolecular fluorescence complementation (BiFC) assay, in which viral proteins and Tm-2^2^ were fused to the N-terminal and C-terminal fragments of YFP (Yn and Yc), respectively. Positive signals were detected when Yc-Tm-2^2^ was co-expressed with Yn-ToBRFV-MP and Yn-TMV-MP, but not with the control (Yn-TuMV-CP; Fig 2H). The expression of recombinant proteins was validated by immunoblotting (Fig 2I). Co-immunoprecipitation assay confirmed the Tm-2^2^ and ToBRFV-MP interaction in *N. benthamiana* (Fig 2J), suggesting Tm-2^2^ does interact with ToBRFV-MP in *planta*.

## Discussion

Available evidence suggests that ToBRFV-MP is the susceptibility determinant, enabling the virus to break down *Tm-2*^*2*^-mediated ER [7, 9, 10]. In this study, we present evidence that ToBRFV-MP retains its ability to trigger a moderate but distinct cell death in the presence of high expression levels of *Tm-2*^*2*^ (Fig 2E), leading to partial resistance to ToBRFV (Fig 1G-1L). This protection is quantitatively dependent on receptor abundance, as elevated Tm-2^2^ expression markedly strengthened viral restriction (Fig 2A-2C), indicating that Tm-2^2^ functions in a dosage-dependent manner [11]. Interestingly, ER conferred by the RCY1 gene to another plant virus also requires its high-level expression [15]. The expression level of *R* genes seems crucial for their antiviral function. Biochemical assays, including trypan blue staining, DAB assays, and ion leakage measurements, support ToBRFV-MP as an *Avr* effector, albeit eliciting a weaker HR, compared to TMV-MP (Fig 2E-2G). The physical association between Tm-2^2^ and ToBRFV-MP *in planta* provides direct molecular evidence that the recognition persists during ToBRFV infection (Fig 2H and 2J). These findings affirm the enduring value of *Tm-2*^*2*^ in tomato resistance breeding and reject the notion that *Tm-2*^*2*^ is an obsolete gene in management of tomamoviruses. Importantly, the novel finding that *Tm-2*^*2*^ resistance to ToBRFV may be enhanced through elevation of *Tm-2*^*2*^ expression offers an alternative avenue to restore *Tm-2*^*2*^ immunity [16].

In addition, we show that ToBRFV-CP, but not TMV-CP, suppresses *Tm-2*^*2*^ transcription (Fig 1B), which may contribute to differential effectiveness of *Tm-2*^*2*^ against TMV and ToBRFV. The partial nuclear localization of CP [13] further supports this possibility. This transcriptional repression likely compromises receptor abundance and limits the amplitude of immune signaling, thereby resulting in a weakened immune activation and subsequent resistance breakdown.

## Materials and methods

Detailed methods and statistical analysis are described in the S1 File.

## Supporting information

S1 File Supplementary materials and methods. (DOCX)

S1 Table List of primers used in this study. (DOCX)

S1 Fig *Tm-2*^*2*^ restricts ToBRFV infection via mechanical inoculation. (A) Phenotypes of *Nicotiana benthamiana* plants mechanically inoculated with ToBRFV. Images were captured at 3 days post inoculation (dpi). White arrows indicate ToBRFV-induced symptoms. Scale bars, 2 cm. (B and C) Accumulation of viral coat protein (CP) and CP transcripts was analyzed by immunoblotting (B) and RT-qPCR analysis (C). The data in (C) were analyzed using unpaired Student’s *t* tests (n = 3; **p < 0.01; ns, not significant). All photographs were taken by the authors.
(TIF)

S2 Fig RT-qPCR analysis of expression of systemic acquired resistance marker genes in response to ToBRFV infection. (A-C) Transcript accumulation of *NbFMO1* (A), *NbICS1* (B), and *NbNPR1* (C) was analyzed in both wild-type and *Tm-2*^*2*^ transgenic *N. benthamiana* plants. Leaf tissues were collected at 3 dpi after mechanical inoculation with either PBS buffer or ToBRFV. The data were analyzed using unpaired Student’s *t* tests (*p < 0.05; **p < 0.01; ns, not significant).
(TIF)

S3 Fig Elevated Tm-2^2^ expression enhances resistance to ToBRFV. (A) Phenotypes of wild-type *N. benthamiana* plants mechanically inoculated with ToBRFV. Images were taken at 3 days post inoculation (dpi). White arrows indicate typical viral symptoms. Scale bars, 2 cm. (B and C) ToBRFV-CP protein and mRNA accumulation was determined by immunoblotting (B) and RT-qPCR analysis (C). The data in (C) were analyzed using unpaired Student’s *t* tests (**p < 0.01; ***p < 0.001; ns, not significant). All photographs were taken by the authors.
(TIF)
