# Supplementary material for: The durable resistance gene Tm-22 remains partially resistant to tomato brown rugose fruit virus
Source: PLoS Pathog. 2026 Jul 17;22(7):e1014380. doi: 10.1371/journal.ppat.1014380 (PMC13379008; doi:10.1371/journal.ppat.1014380)
Supplement: S1 File — (DOCX) [file ppat.1014380.s001.docx]

SHORT REPORTS

**Supporting information**

**The durable resistance gene *Tm-2^2^* remains partially resistant to tomato brown rugose fruit virus**

**Shaokang Zhang, Mark A. Bernards, and Aiming Wang**

**Correspondence: Aiming Wang (aiming.wang@agr.gc.ca)**

**S1 Text. Supplementary materials and methods**

**Virus resources and plant growth conditions**

ToBRFV and TMV-GFP infectious clones were generated as described [1,2]. *Tm-2^2^* transgenic *Nicotiana benthamiana* lines were obtained as described [3]. *Solanum lycopersicum* cultivar Moneymaker plants harboring *Tm-2^2^* were previously described [4]. All plants were grown in an insect-free growth chamber at 24 ± 1 °C under a 16 h/8 h light dark photoperiod. Plants were photographed using a Nikon COOLPIX P500 camera.

**Plasmid construction**

Full length sequences of *Tm-2^2^*, *TMV-MP*, and *TMV-CP* were amplified by Phusion High-Fidelity DNA Polymerase (New England Biolabs, Whitby, ON, Canada) and individually cloned into the entry vector pDONR221 by Gateway BP Clonase (Invitrogen, Burlington, ON, Canada) following the supplier's instructions to generate pDONR221-Tm-2^2^, pDONR221-TMV-MP, and pDONR221-TMV-CP, respectively. The TMV-CP original fragment was synthesized by SinoGenoMax. LTD (Beijing, China). pDONR221-ToBRFV-MP and pDONR221-ToBRFV-CP were generated previously [2].

For transient expression assays, the intermediate pDONR221 clones were transferred into Gateway-compatible binary destination vectors pEarleyGate101 (C-terminal YFP tag), pEarleyGate103 (C-terminal GFP tag), or pBA-Flag-4xMyc (N-terminal Myc and Flag tags) via LR Clonase (Invitrogen) to generate the corresponding plant expression vectors, respectively. For BiFC assays, those entry clones were introduced into modified Gateway-compatible vectors pEarleygate201-Yn or pEarleygate202-Yc [5]. Yn-TuMV-CP and pBA-TuMV-VPG were constructed previously [6,7].

Sequences of plasmids were confirmed by Sanger sequencing (Eurofins Genomics, Louisville, KY, USA). All primers used in this study are listed in List of primers used in this study in Supplementary S3 Text.

**RNA extraction and RT-qPCR**

Total RNA from plant tissues were extracted using the SDS-phenol/chloroform extraction method [8]. Essentially, 0.1 g of leaf tissue was ground in liquid nitrogen, followed by the addition of 600 µL phenol: chloroform: isoamyl alcohol (25:24:1; Invitrogen) and 600 µL of extraction buffer (20 mM Tris-HCl pH 7.8, 1% SDS, 200 mM NaCl, and 5 mM EDTA). After centrifugation at 4 °C, 500 µl of aqueous phase was precipitated with an equal volume of 4 M lithium chloride at -20 °C overnight. RNA pellets were centrifuged and washed twice with 75% and 100% ethanol, respectively. First-strand cDNA was synthesized using 2 µg of total RNA and either Random hexamers (New England Biolabs) or gene-specific primers, with HiScript IV RT SuperMix (Vazyme, Nanjing, China) following the manufacturer's instructions. qPCR analysis was performed using the SensiFAST SYBR No ROX Kit (Meridian Bioscience, Quebec City, QC, Canada) on a Bio-Rad CFX Opus 96 instrument. *N. benthamiana* or tomato *Elongation factor 1α* (*EF-1α*) genes served as the internal control. All primers used for RT-qPCR are listed in S1 Table. All RT-qPCR analysis were repeated at least three times and representative data were selected to generate the plots.

**Agroinfiltration and viral inoculation**

Agroinfiltration assays were performed as described previously [2]. All plasmids were transformed into *Agrobacterium tumefaciens* strains GV3101 via freeze-thawing transformation. After growing in LB broth with 50 μg/mL of kanamycin or 50 μg/mL of Spectinomycin and 25 μg/mL of rifampicin at 28 °C for 16 h, agrobacterium cultures harboring relevant vectors were pelleted and resuspended in infiltration buffer (10 mM MgCl_2_, 100 μM Acetosyringone, and 10 mM MES, pH 5.6). Unless otherwise stated, resuspended cells harboring all transient expression vectors were adjusted to an OD_600_ of 0.3, and those carrying viral infectious clones to OD_600_ of 0.1. After 2 hours of incubation at room temperature, bacterial suspensions were infiltrated into fully expanded leaves of 5-6 leaf stage *N. benthamiana* plants or injected into the stems and cotyledons of 4-week-old tomato plants using a 1 mL sterile syringe. For transient overexpression of Tm-2^2^ prior to viral challenge, suspensions carrying ToBRFV infectious clone were infiltrated into the same leaf area 24 hours after the initial agroinfiltration with the Tm-2^2^-YFP construct.

Mechanical inoculation was carried out essentially as described previously [2]. Briefly, ToBRFV-infected tomato leaf tissue (0.1 g) were ground into a fine powder in liquid nitrogen and then homogenized with 50 ml (1:500, w/v) or 100 ml (1:1000, w/v) 0.01 M PBS buffer (0.01 M Na_2_HPO_4_/KH_2_PO_4_, 0.138 M NaCl, and 0.0027 M KCl, pH 7.5). The homogenate was gently rubbed on the surface of tested plant leaves dusted with carborundum powder. Five minutes after inoculation, the leaves were rinsed using sterilized water. PBS buffer served as a negative control. The 1:500 (w/v) inoculum was used for assays in S2 and S3 Fig in Supplementary S1 Text.

**DAB staining**

3,3′-Diaminobenzidine (DAB) staining was conducted as described previously with minor modifications [9]. Briefly, 1 mg/mL DAB staining solution was prepared by dissolving 50 mg DAB powder in 50 mL deionized water. *N. benthamiana* leaves were collected at 3 dpi, immersed in the staining buffer, and incubated in the dark at room temperature overnight. The stained leaves were then transferred to 95% ethanol and boiled for 10 minutes. The destained leaves were photographed in 75% ethanol. Staining intensity in indicated leaf patches was quantified using ImageJ software (NIH, Bethesda, MD, USA).

**Trypan blue staining**

Trypan blue staining was performed essentially as described, with minor modifications [9,10]. Trypan blue staining solution was prepared in a mixture of 40 mL ethanol, 10 mL lactic acid, 10 mL water-saturated phenol, 10 mL glycerol, 10 mL sterilized water, and 15 mg trypan blue powder. Leaves were immersed in the staining solution, placed in boiling water for 10 minutes, and incubated at room temperature for 6 h. The stained leaves were then destained in 2.5 g/mL chloral hydrate solution overnight with gentle shaking for photography.

**Ion leakage measurement**

The electrolyte ion leakage assay was performed as described previously with minor modifications [10]. Six leaf discs (9 mm in diameter) were excised from infiltrated areas of four plants and floated in 10 mL MilliQ-water for 1 h at room temperature with shaking at 165 rpm. Conductivity values were measured using an Orion Lab Star EC112 Conductivity Bench Meter (Thermo Fisher Scientific, Mississauga, ON, Canada) and recorded as S1. The samples were placed in boiling water for 45 min, followed by shaking at 165 rpm at room temperature for 1 h, and measured to obtain the S2 value. The ratio of S1 to S2 was calculated as electrolyte ion leakage.

**Bimolecular fluorescence complementation (BiFC) assay**

BiFC assays were performed as described previously [11]. Tissues excised from *N. benthamiana* epidermal cells expressing different combinations at 3 dpi were subject to an Olympus FV1200 laser confocal microscopy (Olympus, Richmond Hill, ON, Canada).

**Protein extraction and immunoblot analysis**

Total protein was extracted from 0.1 g of infiltrated leaf patches or viral systemically infected *N. benthamiana* or tomato leaves with 2 x SDS buffer [100 mM Tris (pH 6.8), 4% (w/v) SDS, 20% (v/v) glycerol and 0.2% (w/v) bromophenol blue] according to the procedures described [2]. Protein samples were separated in SDS-polyacrylamide gels and then transferred onto nitrocellulose membranes. Immunoblotting was performed with the following primary rabbit polyclonal antibodies: anti-GFP (1:10000; Proteintech, Rosemont, IL, USA; Cat. # 50430‑2‑AP), anti-C-Myc (1:10000; Sigma-Aldrich, Oakville, ON, Canada; Cat. # C3956), anti-GAPDH (1:5000; Proteintech; Cat. # 10494-1-AP), anti-ToBRFV-CP (1:2000; Agdia, Elkhart, IN, USA; Cat. # SRA 66800). Anti-GFP was also used for YFP-fused protein analysis. After thoroughly washing, membranes were incubated with HRP-conjugated goat anti-rabbit secondary antibody (1:10000; Sigma-Aldrich; Cat. # A6154) and visualized using chemiluminescence following the manufacturer′s protocol (Millipore, Etobicoke, ON, Canada). All immunoblot experiments were repeated at least three times unless otherwise specified. Immunoblot band intensities were quantified using ImageJ software. For each band, the signal intensity was measured as the integrated density and normalized to the corresponding control group, with consistent loading verified by GAPDH or RbcL.

**Co-immunoprecipitation (Co-IP) assay**

Co-IP assays were performed as previously described with minor modifications [11]. In brief, three grams of *N. benthamiana* leaf tissues expressing different combinations were ground in liquid nitrogen and extracted in 6 ml of extraction buffer [25 mM Tris HCl (pH 7.5), 150mM NaCl, 1 mM EDTA, 10% glycerol, 2% w/v polyvinylpolypyrrolidone, 10mM DTT, 1% Tween 20, 1xEDTA-free protease inhibitor cocktail]. After incubating on ice for 30 min, the mixtures were centrifuged at 12,000 rpm at 4 °C for 30 min. The supernatants were passed through a 0.45 µm filter and then incubated with anti-Flag affinity agarose beads (Sigma-Aldrich; Cat. # A2220) for 4 h at 4°C with gentle shaking. The corresponding beads were washed five times with wash buffer [25 mM Tris HCl (pH 7.5), 150mM NaCl, 1 mM EDTA, 10% glycerol, 0.1% Triton X-100] and analyzed by immunoblotting.

**Accession numbers**

The accession numbers of related genes in this study are as follows: *Tm-2^2^* (KY910827.1), ToBRFV genome and encoded *ToBRFV-MP* and *ToBRFV-CP* (PQ592514.1), *TMV-MP* (MK087763.1), *TMV-CP* (NC_001367.1).

**Quantification and statistical analysis**

For RT-qPCR experiments, representative data from at least three biological replicates are presented with mean ± SEM and plotted using GraphPad Prism 7. Statistical significance was determined using unpaired Student's *t*-test between two groups.

**References**

1. Liu Y, Schiff M, Marathe R, Dinesh-Kumar SP. Tobacco *Rar1*, *EDS1* and *NPR1/NIM1* like genes are required for *N*-mediated resistance to tobacco mosaic virus. Plant J. 2002;30(4):415–29. https://doi.org/10.1046/j.1365-313x.2002.01297.x. PMID: 12028572
2. Zhang S, Li Y, Weselowski B, Griffiths JS, Hao X, McCreary CM, et al. Single amino acid change at two conserved residues in tomato brown rugose fruit virus coat protein compromises virion assembly and viral systemic infection. Mol Plant Microbe Interact. 2026;39(1):31–44. https://doi.org/10.1094/MPMI-07-25-0095-R. PMID: 41218208
3. Zhang H, Zhao J, Liu S, Zhang DP, Liu Y. *Tm-2^2^* confers different resistance responses against tobacco mosaic virus dependent on its expression level. Mol Plant. 2013;6(3):971–4. https://doi.org/10.1093/mp/sss153. PMID: 23275490
4. Hak H, Spiegelman Z. The tomato brown rugose fruit virus movement protein overcomes *Tm-2^2^* resistance in tomato while attenuating viral transport. Mol Plant Microbe Interac 2021;34(9):1024–32. https://doi.org/10.1094/MPMI-01-21-0023-R. PMID: 33970669
5. Lu Q, Tang X, Tian G, Wang F, Liu K, Nguyen V, et al. Arabidopsis homolog of the yeast TREX-2 mRNA export complex: components and anchoring nucleoporin. Plant J. 2010;61(2):259–70. https://doi.org/10.1111/j.1365-313X.2009.04048.x. PMID: 19843313
6. Dai Z, He R, Bernards MA, Wang A. The cis-expression of the coat protein of turnip mosaic virus is essential for viral intercellular movement in plants. Mol Plant Pathol. 2020;21(9):1194–211. https://doi.org/10.1111/mpp.12973. PMID: 32686275
7. Li F, Wang A. RNA decay is an antiviral defense in plants that is counteracted by viral RNA silencing suppressors. PLoS Pathog. 2018;14(8):e1007228. https://doi.org/10.1371/journal.ppat.1007228. PMID: 30075014
8. Zhang XY, Zhao TY, Li YY, Xiang HY, Dong SW, Zhang ZY, et al. The conserved proline18 in the polerovirus P3a is important for brassica yellows virus systemic infection. Front Microbiol. 2018;9:613. https://doi.org/10.3389/fmicb.2018.00613. PMID: 29670592
9. Zhang D, Yang X, Wen Z, Li Z, Zhang X, Zhong C, et al. Proxitome profiling reveals a conserved SGT1-NSL1 signaling module that activates NLR-mediated immunity. Mol Plant. 2024;17(9):1369–91. https://doi.org/10.1016/j.molp.2024.07.010. PMID: 39066482
10. Gao Z, Zhang D, Wang X, Zhang X, Wen Z, Zhang Q, et al. Coat proteins of necroviruses target 14-3-3a to subvert MAPKKKα-mediated antiviral immunity in plants. Nat Commun. 2022;13(1):716. https://doi.org/10.1038/s41467-022-28395-5. PMID: 35132090
11. Li F, Zhang C, Li Y, Wu G, Hou X, Zhou X, et al. Beclin1 restricts RNA virus infection in plants through suppression and degradation of the viral polymerase. Nat Commun. 2018;9(1):1268. https://doi.org/10.1038/s41467-018-03658-2. PMID: 29593293
