# Supplementary material for: The durable resistance gene Tm-22 remains partially resistant to tomato brown rugose fruit virus
Source: PLoS Pathog. 2026 Jul 17;22(7):e1014380. doi: 10.1371/journal.ppat.1014380 (PMC13379008; doi:10.1371/journal.ppat.1014380)
Supplement: S1 Table — (DOCX) [file ppat.1014380.s002.docx]

SHORT REPORTS

**Supporting information**

**The durable resistance gene *Tm-2^2^* remains partially resistant to tomato brown rugose fruit virus**

**Shaokang Zhang, Mark A. Bernards, and Aiming Wang**

**Correspondence: Aiming Wang (aiming.wang@agr.gc.ca)**

**S3 Text. List of primers used in this study**

| Primer Name | Primer sequence (5′ to 3′) | Purpose |
| --- | --- | --- |
| attB-Tm-2^2^-F | GGGGACAAGTTTGTACAAAAAAGCAGGCTTCATGGCTGAAATTCTTCTTACATGAG | Construction of Tm-2^2^ entry clone |
| attB-Tm-2^2^-R | GGGGACCACTTTGTACAAGAAAGCTGGGTCTCATTTACTCAGCTTTTTAA |  |
| attB-TMVMP-F | GGGGACAAGTTTGTACAAAAAAGCAGGCTTCATGGCTCTAGTTGTTAAAGG | Construction of TMV-MP entry clone |
| attB-TMVMP-R | GGGGACCACTTTGTACAAGAAAGCTGGGTCAAACGAATCCGATTCGGCGA |  |
| attB-TMVCP-F | GGGGACAAGTTTGTACAAAAAAGCAGGCTTCATGTCTTACAGTATCACTACTC | Construction of TMV-CP entry clone |
| attB-TMVCP-R | GGGGACCACTTTGTACAAGAAAGCTGGGTCAGTTGCAGGACCAGAGGTCCA |  |
| qPCR-NbEF1a-F | TGCTGCAACAAGATGGATGC | RT-qPCR analysis of *NbEF1a* |
| qPCR-NbEF1a-R | CCAGAGATGGGGACAAAGGG |  |
| qPCR-SlEF1a-F | TCCAAAGATGGTCAGACCCGTGAA | RT-qPCR analysis of *SlEF1a* |
| qPCR-SlEF1a-R | ATACCTAGCCTTGGAGTACTTGGG |  |
| qPCR-ToCP-F | CACAATCGCAACTCCATCGC | RT-qPCR analysis of *ToBRFV-CP* |
| qPCR-ToCP-R | ACAGGTTTCCACACTTCGCT |  |
| qPCR-Tm-2^2^-F | CGAGGTTGTTGCACCGATTG | RT-qPCR analysis of *Tm-2^2^* |
| qPCR-Tm-2^2^-R | TTGTTCACTCGGGTCACTGG |  |
| qPCR-NbFMO1-F | CACTGCTTTGCCACATGCTT | RT-qPCR analysis of NbFMO1 |
| qPCR-NbFMO1-R | TTTGCCCTTATCAGCCTCGG |  |
| qPCR-NbICS1-F | GTGTCGGCTCTGCTGTCTTCT | RT-qPCR analysis of NbICS1 |
| qPCR-NbICS1-R | CTGCGTATAGCACGCCAATC |  |
| qPCR-NbNPR1-F | TTACTTCACTGAAACGCCT | RT-qPCR analysis of NbNPR1 |
| qPCR-NbNPR1-R | CACTTCCTTTAATTCCACCT |  |
